# Efficacy of different platforms in detecting *EGFR* mutations using cerebrospinal fluid cell‐free DNA from non‐small‐cell lung cancer patients with leptomeningeal metastases

**DOI:** 10.1111/1759-7714.14866

**Published:** 2023-03-28

**Authors:** Chi‐Lu Chiang, Hsiang‐Ling Ho, Yi‐Chen Yeh, Cheng‐Chia Lee, Hsu‐Ching Huang, Chia‐I Shen, Yung‐Hung Luo, Yuh‐Min Chen, Chao‐Hua Chiu, Teh‐Ying Chou

**Affiliations:** ^1^ Institute of Clinical Medicine National Yang Ming Chiao Tung University Taipei Taiwan; ^2^ School of Medicine National Yang Ming Chiao Tung University Taipei Taiwan; ^3^ Department of Chest Medicine Taipei Veterans General Hospital Taipei Taiwan; ^4^ Department of Pathology and Laboratory Medicine Taipei Veterans General Hospital Taipei Taiwan; ^5^ Department of Biotechnology and Laboratory Science in Medicine National Yang Ming Chiao Tung University Taipei Taiwan; ^6^ Department of Neurosurgery, Neurological Institute, Taipei Veterans General Hospital Taipei Taiwan; ^7^ Taipei Cancer Center Taipei Medical University Hospital, Taipei Medical University Taipei Taiwan; ^8^ Department of Pathology Taipei Medical University Hospital, Taipei Medical University Taipei Taiwan

**Keywords:** cell‐free DNA, cerebrospinal fluid, epidermal growth factor receptor, next‐generation sequencing, non‐small‐cell lung cancer

## Abstract

**Background:**

Cell‐free tumor DNA (ctDNA) obtained through liquid biopsy is useful for the molecular analysis of advanced non‐small‐cell lung cancer (NSCLC). Few studies have directly compared analysis platforms in terms of their diagnostic performance in analyzing ctDNA obtained from the cerebrospinal fluid (CSF) of patients with leptomeningeal metastasis (LM).

**Methods:**

We prospectively analyzed patients with epidermal growth factor receptor (*EGFR*)‐mutant NSCLC who were subjected to CSF analysis for suspected LM. To detect *EGFR* mutations, CSF ctDNA was analyzed using the cobas EGFR Mutation Test and droplet digital polymerase chain reaction (ddPCR). CSF samples from osimertinib‐refractory patients with LM were also subjected to next‐generation sequencing (NGS).

**Results:**

Significantly higher rates of valid results (95.1% vs. 78%, respectively, *p* = 0.04) and *EGFR* common mutation detection (94.3% vs. 77.1%, respectively, *p* = 0.047) were obtained through ddPCR than through the cobas EGFR Mutation Test. The sensitivities of ddPCR and cobas were 94.3% and 75.6%, respectively. The concordance rate for *EGFR* mutation detection through ddPCR and the cobas EGFR Mutation Test was 75.6% and that for *EGFR* mutation detection in CSF and plasma ctDNA was 28.1%. In osimertinib‐resistant CSF samples, all original *EGFR* mutations were detected through NGS. *MET* amplification and *CCDC6‐RET* fusion were demonstrated in one patient each (9.1%).

**Conclusions:**

The cobas EGFR Mutation Test, ddPCR, and NGS appear to be feasible methods for analyzing CSF ctDNA in patients with NSCLC and LM. In addition, NGS may provide comprehensive information regarding the mechanisms underlying osimertinib resistance.

## Graphical abstract

The authors analyzed the cerebrospinal fluid cell‐free tumor DNA from patients with epidermal growth factor receptor (*EGFR*)‐mutant non‐small‐cell lung cancer and leptomeningeal metastasis. They found significantly higher rates of valid results and *EGFR* common mutation detection were obtained through droplet digital polymerase chain reaction (ddPCR) than through the cobas EGFR Mutation Test. The sensitivities of ddPCR and cobas were 94.3% and 75.6%, respectively.

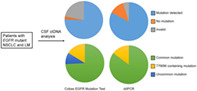

## INTRODUCTION

In recent years, the treatment paradigm for advanced non‐small‐cell lung cancer (NSCLC) has markedly shifted toward precision medicine because of the discovery of driver oncogenes and targeted therapies.^1^, ^2^ Epidermal growth factor receptor (*EGFR*) mutations are the most common oncogenic driver mutations observed in Asian patients with NSCLC.^3^ EGFR tyrosine kinase inhibitors (TKIs) are superior to cytotoxic chemotherapy in terms of efficacy and have become the standard treatment option for patients with *EGFR*‐mutant NSCLC.^4^, ^5^, ^6^ The identification of driver oncogenes from tumor specimens has become a routine task in clinical practice. However, obtaining a sufficient amount of tumor tissue for molecular profiling may be difficult. Liquid (plasma) biopsy is a practical alternative in the case of absent or insufficient tissue samples and can be performed concurrently or sequentially with tumor genotyping in patients with advanced NSCLC.^7^, ^8^

Patients with *EGFR*‐mutant NSCLC have a higher risk of central nervous system (CNS) involvement during the treatment course than do patients with *EGFR* wild‐type NSCLC.^9^ Among the neurological complications of NSCLC, leptomeningeal metastasis (LM) is the most devastating form of CNS metastasis, with a median overall survival (OS) <1 year.^10^ The incidence of LM in patients with *EGFR*‐mutant NSCLC was reported to be 9.4%.^11^ However, obtaining tumor specimens from intracranial lesions is challenging, particularly in patients with LM, because biopsy of the leptomeninges is not clinically feasible. Therefore, the use of cerebrospinal (CSF) for liquid biopsy to access circulating tumor cells or cell‐free tumor DNA (ctDNA) is a new approach for genotyping intracranial lesions in patients with NSCLC and LM.^12^ Studies have indicated the clinical usefulness of detecting sensitive and resistant *EGFR* mutations in CSF ctDNA.^13^, ^14^

The rates of mutation detection in patients with *EGFR*‐mutant NSCLC vary across sequencing methods. Droplet digital polymerase chain reaction (ddPCR), which combines microfluidic technology with quantitative PCR, is an ultra‐sensitive assay for measuring absolute mutation alleles that has been used to detect low‐abundance nucleic acids in clinical practice.^15^ For the detection of plasma *EGFR* mutations, the sensitivity of ddPCR is higher than that of the amplification refractory mutation system (ARMS) PCR.^16^, ^17^ However, studies directly comparing the mutation detection rate of different sequencing platforms using CSF ctDNA are scarce. Therefore, in this study, we compared multiple PCR methods in terms of their diagnostic performance in analyzing CSF ctDNA and explored their clinical relevance.

## METHODS

### Study design and patients

We prospectively analyzed patients with *EGFR*‐mutant NSCLC who were subjected to CSF analysis for suspected LM between August 2020 and November 2021. Data regarding the patients' clinical characteristics were collected, including age, sex, Eastern Cooperative Oncology Group (ECOG) performance status, primary or metastatic tumor's *EGFR* mutation status, history of systemic treatment before and after CSF sampling, brain magnetic resonance imaging (MRI) findings, CSF analysis reports, and survival status. The European Association of Neuro‐Oncology (EANO)–European Society for Medical Oncology (ESMO) guideline was used to diagnose LM in this study.^18^ In brief, positive CSF cytology indicated “confirmed LM,” classical neurological image findings (i.e., MRI), typical clinical signs indicated “probable LM,” and only typical neurological symptoms indicated “possible LM.” This study was approved by the Institutional Review Board of Taipei Veterans General Hospital, Taiwan (approval number: 2020‐07‐008C) and conducted in accordance with the ethical principles of Declaration of Helsinki.

### Sample preparation and ctDNA isolation

Lumbar puncture was performed to diagnose LM and CSF was collected. CSF collection was also collected through a ventriculoperitoneal shunt. Then 5–8 mL of CSF was reserved for cytological assessments, and the residual sample was sent for cell count and biochemistry analyses. The CSF sample was centrifuged at 400*g* for 5 min at 4°C, and the pellet was collected and sent for cytological assessments, which were performed by experienced cytologists. The supernatant was recentrifuged at 2000*g* for 10 min at 4°C, and the secondary supernatant was stored at −80°C until ctDNA extraction. CSF ctDNA was extracted using the cobas DNA Sample Preparation Kit (Roche Molecular Systems) according to the manufacturer's instructions.

To obtain plasma ctDNA, venipuncture was performed to collect peripheral blood samples (10 mL), which were kept in K2 EDTA tubes at the time of CSF sampling and processed within 1 h, when feasible. The tubes were centrifuged twice at 2000*g* for 10 min at 4°C, and ctDNA was extracted from 2 mL of plasma sample using the QIAamp Circulating Nucleic Acid Kit (Qiagen) according to the manufacturer's instructions.

### *EGFR* mutation analysis using the cobas *EGFR* Mutation Test

The ctDNA extracted from CSF and plasma samples was analyzed using the cobas *EGFR* Mutation Test (version 2; Roche Diagnostics), which is a mutant allele‐specific real‐time PCR. This test was performed to detect 42 types of *EGFR* mutations in exons 18–21. The results were interpreted according to the manufacturer's instructions. “Mutation detected” or “no mutation” indicated the detection of at least one mutation or no mutation in the targeted *EGFR* region, respectively. “Invalid” signified that the extracted DNA was of insufficient quantity or quality to repeat the amplification and detection steps in each sample.

### *EGFR* mutation analysis using ddPCR

Each PCR sample was partitioned into 20 000 droplets and analyzed using a QX200 Droplet Digital PCR system (Bio‐Rad Laboratories). Following amplification, each droplet was scored as positive or negative after the detection of the fluorescence signal emitted by the target sequence. Poisson statistical analysis was performed for the absolute quantification of the target sequence. To analyze *EGFR* mutations, in addition to a reference assay, TaqMan *EGFR* T790M mutation assay, *EGFR* Exon 19 deletions assay, *EGFR* L858R mutation assay, and *EGFR* C797S mutation assay (Invitrogen Life Technologies) were performed. In brief, 20 μL of a ddPCR reaction mix containing 2X Master Mix (Bio‐Rad Laboratories), 20X primer, and TaqMan Probe mix (Applied Biosystems Life Technologies) in addition to the DNA template was prepared and subjected to emulsification using the QX200 droplet generator. The emulsified samples were then transferred into 96‐well plates for PCR, and the PCR products were loaded into a droplet reader (QX200 Droplet Digital System; Bio Rad Laboratories) for analysis of mutations through QuantaSoft (version 1.7.4.0917; Bio‐Rad Laboratories) The result was interpreted as positive if the mutant copy number was ≥3 in the ddPCR assays. Fractional abundance (FA), which indicates the abundance of mutant DNA alleles in a wild‐type background, was also evaluated.^19^

### Next‐generation sequencing

In osimertinib‐resistant patients with LM, the extracted ctDNA from CSF was sent for Oncomine Lung cfDNA Assay if residual samples were available. The MagMAX Cell‐Free DNA Isolation Kit was used for ctDNA isolation. We applied the Ion Chef System and Ion S5 XL sequencing system for template preparation and next‐generation sequencing (NGS), respectively. Eleven genes (*ALK*, *BRAF*, *EGFR*, *ERBB2*, *KRAS*, *MAP2K1*, *MET*, *NRAS*, *PIK3CA*, *ROS1*, and *TP53*) with ≥150 hotspots were analyzed. Ion Reporter was used for postsequencing analysis.

### Statistical analysis

Patient characteristics were summarized using descriptive statistics. Categorical variables are presented as numeric and percentage values, whereas continuous variables are presented as median and range values. Chi‐square and Fisher's exact test were used to analyze the correlations between detectable mutations in CSF ctDNA and the patients' demographic characteristics. The sensitivity, specificity, positive predictive value, negative predictive value (NPV), and accuracy of each platform was measured according to the relevant formulas. The Kaplan–Meier method was used to plot survival curves, and the log‐rank test was used to perform between‐group comparisons. *p* < 0.05 was considered statistically significant. OS was calculated as the interval between the date of CSF sampling and that of death or most recent follow‐up. All analyses were performed using SPSS for Windows (version 22.0; IBM Corporation).

## RESULTS

### Patient characteristics

This study enrolled 40 patients; 30 patients and seven patients were diagnosed to have confirmed LM or probable LM, respectively. The median age of patients with LM was 64.5 years (range 37.3–99.4 years). Most were women (67.6%), never smoked (75.7%) and had an ECOG performance score of 0–1 (51.4%). Exon 21 L858R mutation (54.1%) was found to be the most common *EGFR* mutation in our study, followed by exon 19 deletion (32.4%). Fourteen patients (37.8%) received whole‐brain radiotherapy (WBRT) before CSF analysis. Of all the patients, >90% had received at least one targeted therapy before CSF analysis; osimertinib was the most commonly used EGFR‐TKI (32.4%). Regarding the brain MRI images, the most common finding was parenchymal metastasis (81.1%), followed by leptomeningeal enhancement (78.4%) and ventricular dilation (64.9%). The median duration from initial NSCLC diagnosis to CSF sampling was 30.9 months (range 0–103.2 months). The most common method used for CSF collection was lumbar puncture (78.4%); CSF sampling was performed more than once for three patients. The patient characteristics are summarized in Table 1.

**TABLE 1:** Clinical characteristics of all LM patients (*n* = 37)

Characteristics	Number (%)
Gender	
Male	12 (32.4)
Female	25 (67.6)
Age at CSF sampling (year)	
Median (range)	64.5 (37.3–99.4)
History of smoking	
Never smoker	28 (75.7)
Ever smoker	9 (24.3)
ECOG PS at CSF sampling	
0–1	19 (51.4)
2	18 (48.6)
EGFR mutation status at diagnosis	
Exon 19 deletion	12 (32.4)
G719X	3 (8.1)
L858R	20 (54.1)
L858R + T790M	1 (2.7)
L861Q	1 (2.7)
CSF sampling	
1 time	34 (91.9)
2 times	2 (5.4)
3 times	1 (2.7)
History of WBRT before CSF sampling	
Yes	14 (37.8)
No	23 (62.2)
Lines of EGFR‐TKI before CSF sampling	
0	2 (5.4)
1	17 (45.9)
2	12 (32.4)
3	6 (16.2)
Last EGFR‐TKI before CSF sampling	
Gefitinib	2 (5.4)
Erlotinib	9 (24.3)
Afatinib	11 (29.7)
Osimertinib	12 (32.4)
Almonertinib	1 (2.7)
TKI naïve	2 (5.4)
Extra‐cranial disease status at CSF sampling	
LM at lung cancer diagnosis	1 (2.7)
Stable disease	29 (78.4)
Disease progression	7 (18.9)
Brain MRI finding	
Parenchymal metastasis	30 (81.1)
Leptomeningeal enhancement	29 (78.4)
Dilated ventricular system	24 (64.9)
Time from diagnosis to first CSF sampling	
Months (median, range)	30.9 (0–103.2)
Method of CSF sampling	
Lumbar puncture	29 (78.4)
From VP shunt	8 (21.6)
CSF cytology report	
Adenocarcinoma	30 (81.1)
Atypical cells	3 (8.1)
Negative for malignant cell	4 (10.8)

*Abbreviations*: CSF, cerebrospinal fluid; ECOG PS, Eastern Cooperative Oncology Group Performances Status; EGFR, epidermal growth factor receptor; LM, leptomeningeal metastasis; MRI, magnetic resonance imaging; TKI, tyrosine kinase inhibitor; VP, ventriculoperitoneal shunt; WBRT, whole‐brain radiation therapy.

### Diagnostic performance of the cobas EGFR Mutation Test and ddPCR

Supporting Information Table S1 summarizes the analysis results of the 41 CSF samples obtained from patients with confirmed or probable LM (*n* = 37). The median white blood cell (WBC) count in the CSF was 3/cumm, and the median level of protein was 69.6 mg/dL. A significantly higher rate of valid results was obtained through ddPCR than through the cobas EGFR Mutation Test (95.1% vs. 78%, respectively, *p* = 0.04; Supporting Information Table S2).

**FIGURE 1:**
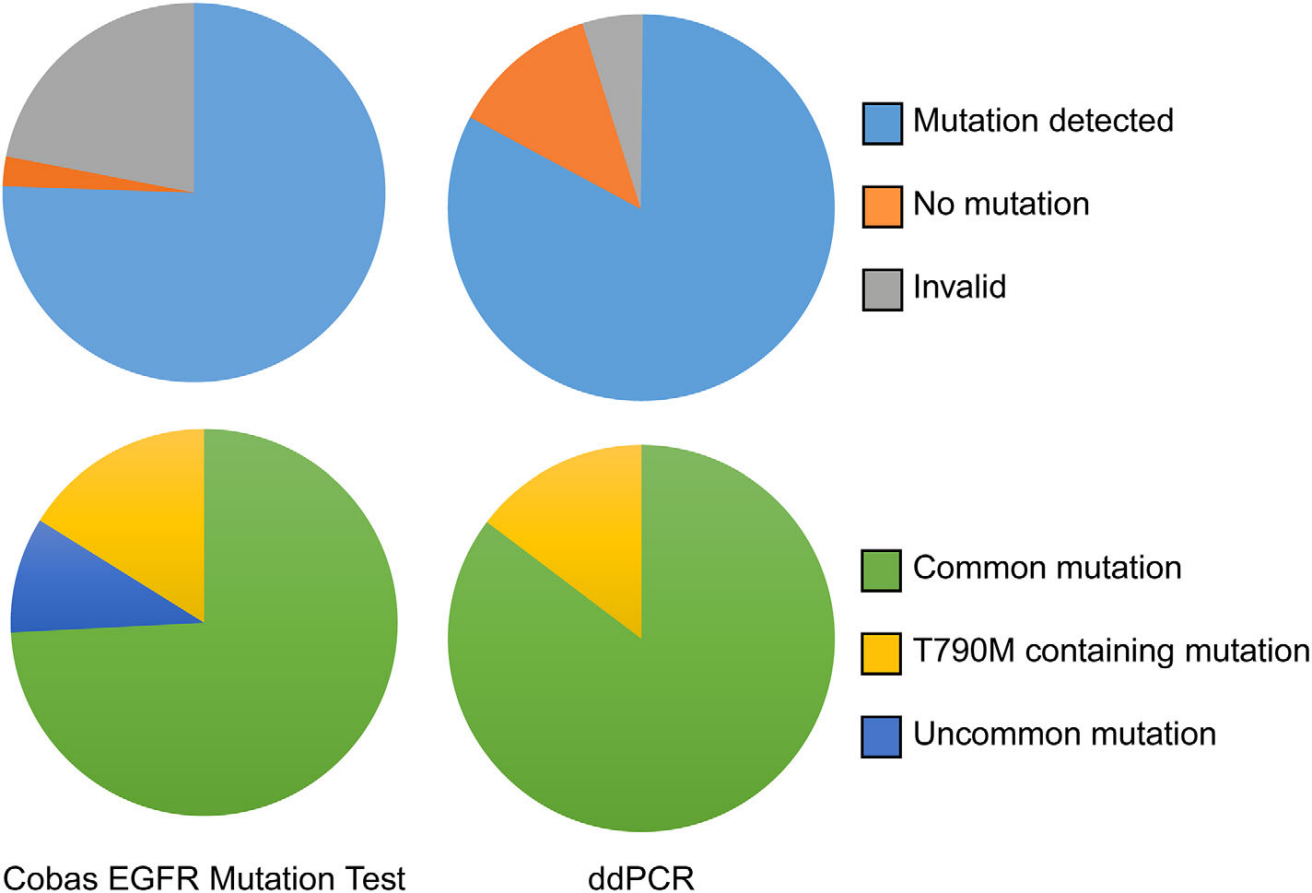
Rates of mutation detection obtained using the cobas EGFR Mutation Test and ddPCR. ddPCR, droplet digital polymerase chain reaction; EGFR epidermal growth factor receptor

The detection rate of *EGFR* mutation in the CSF ctDNA analyzed using the cobas EGFR Mutation Test was 75.6% (31/41). This value was 81.1% (30/37) in patients with abnormal cytology results. However, using ddPCR, the mutation detection rates were 82.9% (34/41) and 86.5% (32/37) in the overall and cytological abnormal samples, respectively (Figure 1). For patients carrying common *EGFR* mutations (exon 19 deletion and exon 21 L585R mutation) with LM, the rate of mutation detection was significantly higher with ddPCR than with the cobas EGFR Mutation Test (94.3% vs. 77.1%, respectively, *p* = 0.047). The diagnostic performance of each platform is presented in Table 2. Furthermore, ddPCR exhibited higher sensitivity (94.3%), NPV (50%), and accuracy (94.6%) compared with the cobas EGFR Mutation Test and CSF cytology.

**TABLE 2:** Diagnostic performance of different platforms

Diagnostic parameters	Cobas EGFR Mutation Test (%)	ddPCR (%)	CSF‐cytology (%)
Sensitivity	75.6	94.3	78.1
Specificity	100	100	100
PPV	100	100	100
NPV	23.1	50	25
Accuracy	77.3	94.6	79.6

*Abbreviations*: CSF, cerebrospinal fluid; ddPCR, droplet digital polymerase chain reaction; EGFR, epidermal growth factor receptor; NPV, negative predictive value; PPV, positive predictive value.

Overall, the concordance rate for *EGFR* mutations detected using the cobas EGFR Mutation Test and ddPCR was 75.6% (31/41; Table 3), rising to 82.9% (29/35) if patients with uncommon mutations were excluded (Supporting Information Table S3). Six patients returned invalid results in cobas but were revealed to have detectable *EGFR* mutations in ddPCR testing. The clinical characteristics of these six patients are listed in Supporting Information Table S4. Five patients (13.5%, 5/37) had *EGFR* T790M mutation, which were detected through both the cobas EGFR Mutation Test and ddPCR. The mutation profiles of all enrolled patients are illustrated in Supporting Information Figure S1.

**TABLE 3:** Comparison of *EGFR* mutation status from CSF ctDNA using the cobas EGFR Mutation Test and ddPCR

	Cobas EGFR Mutation Test							
ddPCR	E19D	E19D/T790M	L858R	L858R/T790M	Uncommon mutation^a^	Invalid	No mutation	Total
E19D	9	0	0	0	0	2	0	11
E19D/T790M	0	1	0	0	0	0	0	1
L858R	0	0	14	0	0	4	0	18
L858R/T790M	0	0	0	3	0	0	0	3
T790M	0	0	0	0	1^b^	0	0	1
Invalid	0	0	0	0	0	2	0	2
No mutation	0	0	0	0	3	1	1	5
Total	9	1	14	3	4	9	1	41

*Abbreviations*: CSF, cerebrospinal fluid; ddPCR, droplet digital polymerase chain reaction; EGFR, epidermal growth factor receptor; E19D, exon 19 deletions.

^a^ Uncommon mutation: G719X, L861Q.

^b^ This sample had EGFR L861Q + T790M mutation.

The median FA of *EGFR* mutations detected through ddPCR was 53.7% (range 0.15–94%); this result did not correlate with the WBC count or protein level in the CSF. Similarly, no correlation was observed between the presence of adenocarcinoma cells in the CSF and high FA (*p* = 0.253).

### Comparison of *EGFR* mutations in CSF and plasma ctDNA

Thirty‐two patients received plasma ctDNA analysis at the time of CSF sampling, and most had no detectable *EGFR* mutation in plasma (65.6%, 21/32). Among the patients with detectable *EGFR* mutations in plasma ctDNA, 21.9% of cases (7/32) had L858R and 12.5% (4/32) had exon 19 deletion. We detected T790M mutation in only three cases (9.4%). The concordance rate for *EGFR* mutations detected in CSF ctDNA using ddPCR and plasma ctDNA was 28.1% (9/32; Table 4).

**TABLE 4:** Comparison of plasma *EGFR* mutation results and the ddPCR test of CSF ctDNA in leptomeningeal metastasis patients harboring *EGFR* common mutations (*n* = 32).

	Plasma *EGFR* mutation test					
CSF ctDNA ddPCR	E19D	E19D/T790M	L858R	L858R/T790M	No mutation	Total
E19D	2	1	0	0	5	8
E19D/T790M	0	0	0	0	1	1
L858R	0	0	4	1	10	15
L858R/T790M	0	0	2	0	1	3
No mutation	0	0	0	0	3	3
Invalid	0	1	0	0	1	2
Total	2	2	6	1	21	32

Abbreviations: CSF, cerebrospinal fluid; ctDNA, cell‐free tumor DNA; ddPCR, droplet digital polymerase chain reaction; EGFR, epidermal growth factor receptor; E19D, exon 19 deletion.

### ctDNA profiling of Osimertinib‐resistant CSF samples

**FIGURE 2:**
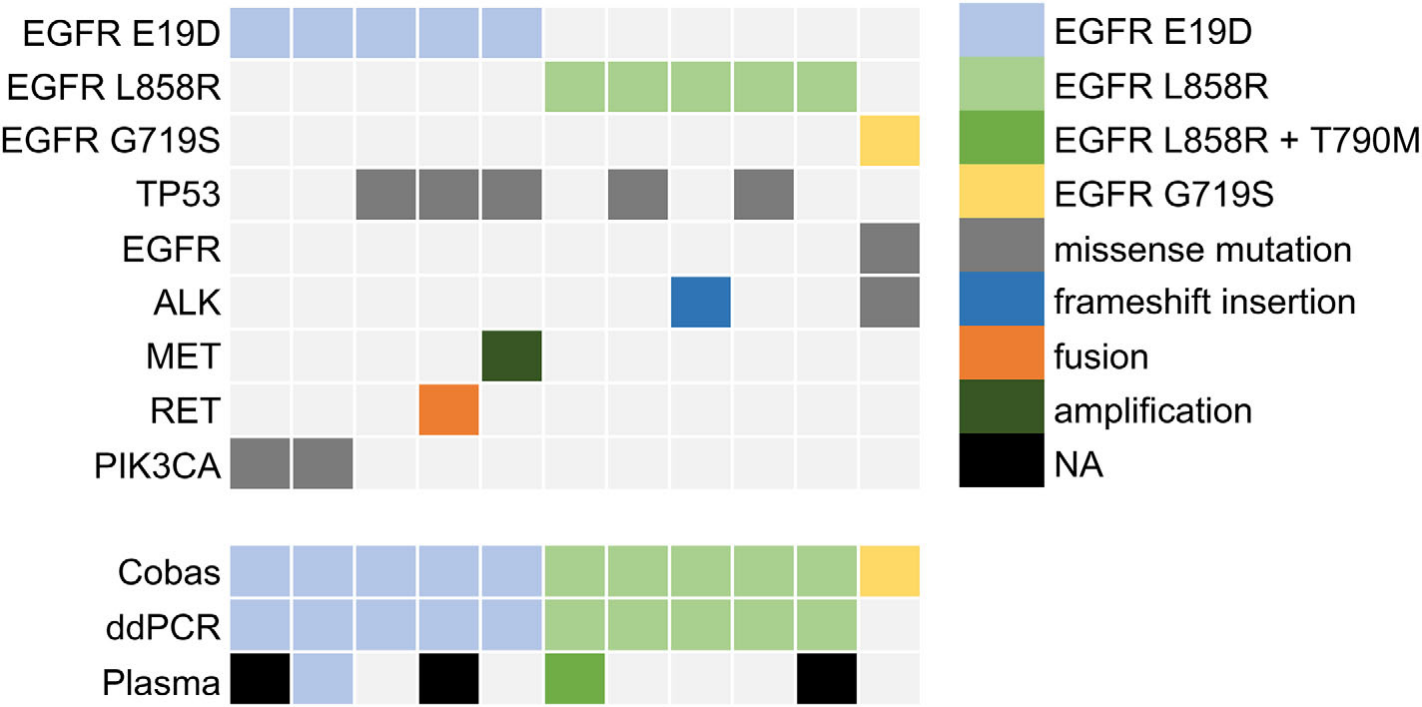
Next‐generation sequencing analysis of cerebrospinal fluid cell‐free tumor DNA extracted from patients receiving osimertinib. ALK, anaplastic lymphoma kinase; ddPCR, droplet digital polymerase chain reaction; E19D, exon 19 deletion; EGFR, epidermal growth factor receptor; NA, not available

The CSF samples of 11 patients who developed LM progression after osimertinib use were sent for cobas *EGFR* Mutation Test, ddPCR, and NGS testing using the Oncomine Lung cfDNA Assay (Figure 2). All patients had *EGFR* mutations detectable using the cobas EGFR Mutation Test and NGS. One patient (9%) exhibited *EGFR* L747S mutation. Following *EGFR* mutations, *TP53* mutation was the second most common co‐mutation detected through NGS (45.5%, 5/11). *MET* amplification and *CCDC6‐RET* fusion, which were reported to be associated with osimertinib resistance, were each observed in one patient. The treatment history and serial genetic profiling results of patient VGH034 are presented in Supporting Information Figure S2. This patient also had systemic progression at the time of CSF sampling. *CCDC6‐RET* fusion was also detected through plasma ctDNA analysis using FoundationOne LiquidCDx.

### OS after LM

**FIGURE 3:**
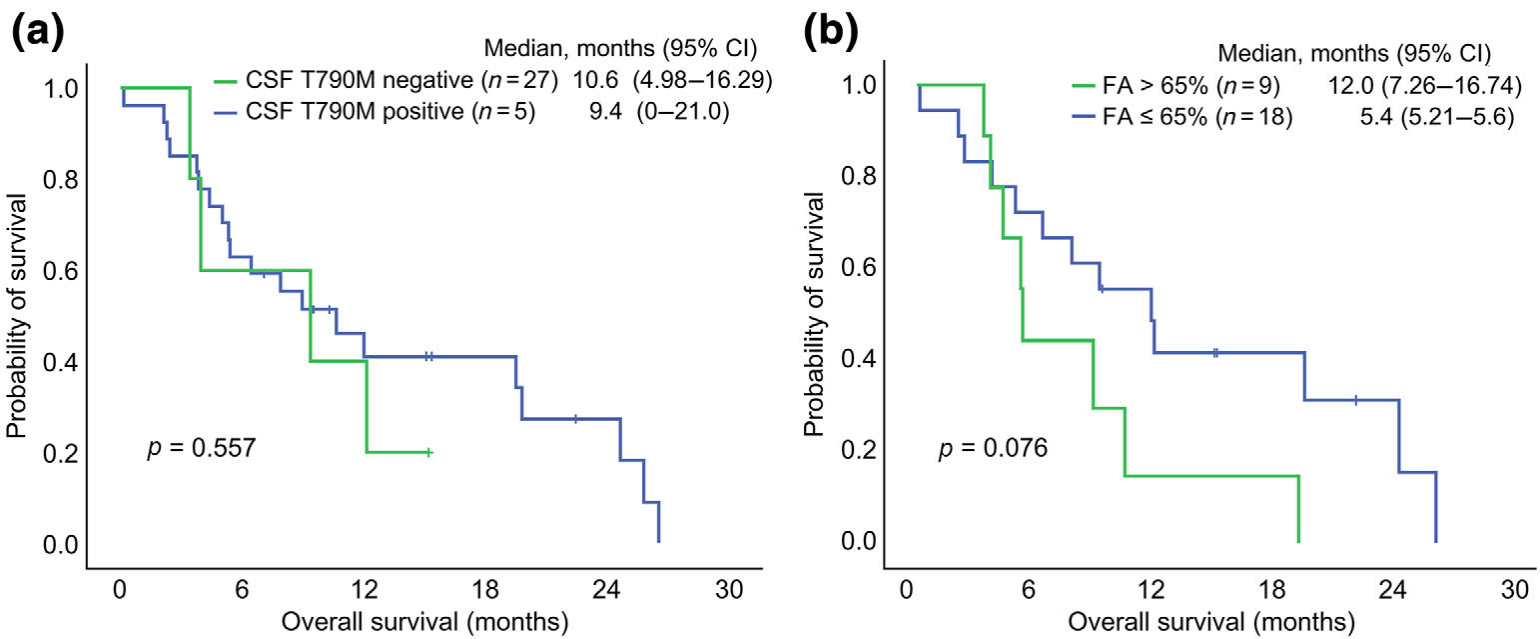
Overall survival in patients receiving osimertinib after leptomeningeal metastasis stratified by the presence of a T790M mutation (a) in the CSF and the FA of EGFR mutations (b). CI, confidence interval; CSF, cerebrospinal fluid; FA, fractional abundance

In this study, 32 (80%) patients received osimertinib after CSF sampling and 12 (30%) patients received WBRT after LM. The median OS was 7.5 months (95% confidence interval 3.54–11.46). The OS was numerically higher in patients receiving osimertinib than in those not receiving it (9.0 vs. 3.5 months, respectively, *p* = 0.253). In the patients receiving osimertinib after LM, the presence of a T790M mutation did not affect the OS after osimertinib use. Patients with a higher FA of EGFR mutation (>65%) had inferior OS after osimertinib use compared with those with lower FA, but the difference is not statistically significant (12 vs. 5.4 months, respectively, *p* = 0.076; Figure 3).

## DISCUSSION

In patients with NSCLC and LM, molecular profiling is crucial for diagnosis and treatment decisions. Reports comparing the different sequencing platforms used for CSF ctDNA are limited. In this study, we demonstrated that CSF ctDNA analysis using the cobas EGFR Mutation Test, ddPCR, and NGS was feasible. Compared with the cobas EGFR Mutation Test, ddPCR had a significantly higher rate of valid results and a higher rate of *EGFR* mutation detection. In addition to *EGFR* sensitizing mutation, the genetic alteration of resistance could be detected through NGS following targeted therapy.

In this study, the rates of *EGFR* mutations detected in CSF ctDNA through the cobas EGFR Mutation Test were 75.6% and 81.1% in the overall patient samples and abnormal cytology samples, respectively. This finding is consistent with that in our previous report.^13^ The rate of *EGFR* mutation detected in CSF ctDNA through ddPCR was 82.9%, which was similar to corresponding results reported in other studies conducted using the same technique.^20^, ^21^ Using primary tumor testing results as a reference, Xu et al. reported similar sensitivity for ddPCR and the ARMS in relation to CSF ctDNA analysis (95% and 89.2%, respectively).^22^ The sensitivity of ddPCR in our study was 94.3%, which was higher than that of the cobas EGFR Mutation Test and cytology testing. Notably, the *EGFR* mutation detection rate in plasma in our study was only 28.1%. These findings further support the use of CSF ctDNA alongside cytological assessments for the diagnosis and treatment of patients with NSCLC and LM.

Acquired resistance is inevitable after the application of targeted therapy. The most common mechanism underlying resistance to first‐ and second‐generation EGFR‐TKIs is an acquired T790M mutation in *EGFR*, which has an incidence rate of 52.8–63%.^15^, ^23^ Despite the use of different platforms, the percentage of T790M mutation is lower in CSF ctDNA analysis.^13^, ^14^, ^20^ Osimertinib, a third‐generation EGFR–TKI that overcomes the resistance conferred by the T790M mutation, has a higher CNS penetration rate than do other EGFR‐TKIs and has exhibited promising intracranial activity in prospective studies.^10^, ^24^ In addition, osimertinib has demonstrated efficacy in patients with *EGFR*‐mutant LM.^25^, ^26^ In our study, 30% of the patients underwent CSF sampling after osimertinib treatment. The percentages of T790M mutation in the CSF ctDNA of all patients and those treated with osimertinib were 13.5% and 8.3%, respectively. The *EGFR* T790M mutation was detected in the patients' CSF samples by using ddPCR and the cobas EGFR Mutation Test. The presence of a T790M mutation did not affect the survival outcome after LM in patients receiving osimertinib. Because the number of patients harboring *EGFR* T790M mutation in their CSF was small in our cohort, further studies are required to compare the efficacy of different sequencing methods in detecting *EGFR* T790M mutation and to evaluate its prognostic significance.

Circulating ctDNA is associated with tumor volume, and a high‐variant allele frequency of ctDNA may lead to inferior survival in patients with metastatic malignancies.^27^, ^28^ However, few studies have focused on the association between the prognosis of patients with LM and the percentage of mutant DNA alleles in their CSF. In our study, patients with a higher FA of *EGFR* mutations were likely to have inferior outcomes after osimertinib use. However, the difference was nonsignificant. Further studies are warranted to validate the prognostic value of ctDNA level in patients' CSF.

The resistance mechanisms of osimertinib, which can be divided into *EGFR*‐dependent and *EGFR*‐independent, are heterogeneous and complex.^29^, ^30^ Eleven patients in our cohort underwent CSF ctDNA analysis through NGS. The detection of *EGFR* mutation was 100%, and no patient had detectable *EGFR* T790M mutation in CSF following osimertinib treatment. Using targeted sequencing, Zheng et al. observed that 21.7% of osimertinib‐refractory patients maintained their T790M mutation.^31^ *EGFR* C797X mutation is a major resistance mutation that emerges following osimertinib treatment that was also observed during CSF ctDNA analysis in a previous study.^32^ However, no patient had detectable C797X mutation in our study. One patient exhibited *MET* amplification and another exhibited *RET* fusion following osimertinib treatment. *MET* copy number gains are a relatively common resistance mechanism in patients with NSCLC and LM.^14^, ^33^ Furthermore, oncogenic fusions were reported to be a bypass pathway of osimertinib resistance, with an incidence range from 1% to 10%.^30^ *CCDC6‐RET* fusion was detected in both the CSF and plasma ctDNA of one patient with progressive LM in our study. It is rare to observe *CCDC6‐RET* fusion in CSF in the context of resistance to osimertinib. However, due to the fact that the patient also exhibited systemic progression, it is challenging to ascertain whether this oncogenic fusion is an intracranial resistance mechanism to osimertinib. Combined *EGFR* and *RET* inhibition was reported to be an effective treatment strategy for such patients^34^; however, our patient developed rapid tumor progression and died before novel targeted treatment could be administered. Together, NGS of CSF ctDNA may provide more information for patients who have progressive LM after CNS‐penetrant TKI therapy. More studies are required to elucidate the resistance mechanism of intracranial lesions following targeted therapy.

This study had some limitations. First, no other genetic alterations were investigated using the CSF and plasma samples, hence we could not construct the comprehensive mutation profiles of the patients with EGFR‐mutant NSCLC and LM. NGS of CSF ctDNA is a feasible method for detecting resistance mutation after new‐generation targeted therapy.^14^, ^35^ However, to the best of our knowledge, most countries lack a commercially available sequencing platform. Second, the mechanisms underlying osimertinib resistance could not be elucidated because of the small number of samples. Nevertheless, our study provided meaningful results and can be used to establish a reasonable strategy for *EGFR* mutation analysis of CSF in populations with a relatively high incidence of *EGFR‐*mutant NSCLC.

In conclusion, our study demonstrated that the cobas EGFR Mutation Test, ddPCR, and NGS are all feasible methods for CSF ctDNA analysis in patients with NSCLC and LM. Our results revealed that the sensitivity of ddPCR is higher than that of the cobas EGFR Mutation Test. NGS may provide more comprehensive information on resistance mechanisms following osimertinib treatment.

## AUTHOR CONTRIBUTIONS

Conceptualisation: CLC, TYC

Data collection, experiment, and analysis: CLC, HLH, YCY, CCL, HCH, CIS, YHL, CHC

Statistics: CLC, HLH

First draft preparation: CLC, CHC, YMC

Review and final approval: all authors.

## CONFLICT OF INTEREST STATEMENT

Chi‐Lu Chiang has received honoraria from AstraZeneca, Boehringer Ingelheim, Pfizer, and Roche. Yung‐Hung Luo has received honoraria from AstraZeneca, Boehringer Ingelheim, and Pfizer. Yuh‐Min Chen has received honoraria from Boehringer Ingelheim, Eli Lilly, Roche/Genentech/Chugai, MSD, Pfizer, Novartis, BMS, Ono Pharmaceutical, AstraZeneca, and Takeda Oncology, and served as an advisor for Boehringer Ingelheim, Eli Lilly, Roche/Chugai, MSD, AstraZeneca, and Takeda Oncology. Chao‐Hua Chiu has received honoraria from AstraZeneca, Boehringer Ingelheim, Pfizer, and Roche. The other authors declare no conflict of interest that might be relevant to the contents of this manuscript.

## Supporting information


**Data S1**. Supporting Information.
