# Supplementary material for: Efficacy of different platforms in detecting EGFR mutations using cerebrospinal fluid cell‐free DNA from non‐small‐cell lung cancer patients with leptomeningeal metastases
Source: Thorac Cancer. 2023 Mar 28;14(14):1251–9. doi: 10.1111/1759-7714.14866 (PMC10175033; doi:10.1111/1759-7714.14866)

Supplemental table 1 CSF analysis of patients with probable or confirmed leptomeningeal metastasis using Cobas EGFR Mutation Test (41 samples)

|  | All | Valid *EGFR* mutation result  (n = 32) | Invalid *EGFR* mutation result  (n = 9) | P value |
| --- | --- | --- | --- | --- |
| WBC /cumm, median  (range) | 3  (0-120) | 4  (0-45) | 2  (0-120) | 0.051 |
| Protein (mg/dl), median (range) | 69.6  (7.7-369.2) | 76.3  (7.7-369.2) | 57.7  (28.1-179.9) | 0.169 |
| Glucose (mg/dl), median (range) | 51  (7-129) | 51  (7-106) | 51  (27-129) | 0.292 |
| Cytology report  Adenocarcinoma  Atypical cells  Negative | 32 (80)  5 (12.2)  4 (9.8) | 27 (84.3)  3 (9.4)  2 (6.2) | 5 (55.6)  2 (22.2)  2 (22.2) | 0.087* |

* Adenocarcinoma vs. others

CSF, cerebrospinal fluid

Supplemental Table 2 *EGFR* mutation profile from CSF ctDNA using cobas *EGFR* Mutation Test and the ddPCR in LM cases (41 samples)

| EGFR mutation | Cobas EGFR Mutation Test Number (%) | ddPCR  Number (%) |
| --- | --- | --- |
| Exon 19 deletion | 9 (22) | 11 (26.8) |
| Exon 19 deletion + T790M | 1 (2.4) | 1 (2.4) |
| L858R | 14 (34.1) | 18 (43.9) |
| L858R + T790M | 3 (7.3) | 3 (7.3) |
| T790M |  | 1 (2.4) |
| Uncommon mutation  G719X*  L861Q + T790M | 3 (7.3)  1 (2.4) |  |
| No mutation | 1 (2.4) | 5 (12.2) |
| Invalid | 9 (22) | 2 (4.9) |

*One case with G719X + exon 19 deletion

ddPCR, droplet digital polymerase chain reaction

LM, leptomeningeal metastasis

Supplemental Table 3 Comparison of *EGFR* mutation status from CSF ctDNA using ARMS and the ddPCR (excluding patients with EGFR uncommon mutation)

|  | Cobas EGFR Mutation Test | | | | | | |
| --- | --- | --- | --- | --- | --- | --- | --- |
| ddPCR | E19D | E19D/  T790M | L858R | L858R/T790M | Invalid | No mutation | Total |
| E19D | 9 | 0 | 0 | 0 | 2 | 0 | 11 |
| E19D/  T790M | 0 | 1 | 0 | 0 | 0 | 0 | 1 |
| L858R | 0 | 0 | 14 | 0 | 4 | 0 | 18 |
| L858R/  T790M | 0 | 0 | 0 | 3 | 0 | 0 | 3 |
| Invalid | 0 | 0 | 0 | 0 | 1 | 0 | 1 |
| No  mutation | 0 | 0 | 0 | 0 | 0 | 1 | 1 |
| Total | 9 | 1 | 14 | 3 | 7 | 1 | 35 |

Abbreviations: E19D, exon 19 deletions

ddPCR, droplet digital polymerase chain reaction

Supplemental Table 4 Patients with disconcordant results

| Subject number | Tumor EGFR mutation | CSF cytology | CSF ctDNA by Cobas EGFR Mutation Test | CSF ctDNA by ddPCR | WBC count, CSF (/cumm) | CSF protein level  (mg/dl) |
| --- | --- | --- | --- | --- | --- | --- |
| 3 | L858R | adenocarcinoma | invalid | L858R | 1 | 36.9 |
| 17 | L858R | adenocarcinoma | invalid | L858R | 4 | 147.6 |
| 27 | E19Del | adenocarcinoma | invalid | E19Del | 3 | 28.1 |
| 33 | L858R | negative | invalid | L858R | 0 | 60.4 |
| 35 | L858R | adenocarcinoma | invalid | L858R | 1 | 40.5 |
| 40 | E19Del | adenocarcinoma | invalid | E19Del | 2 | 179.9 |

CSF, cerebrospinal fluid; ddPCR, droplet digital polymerase chain reaction

Supplemental Figure 1 *EGFR* Mutation Profile of Patients With Leptomeningeal metastasis


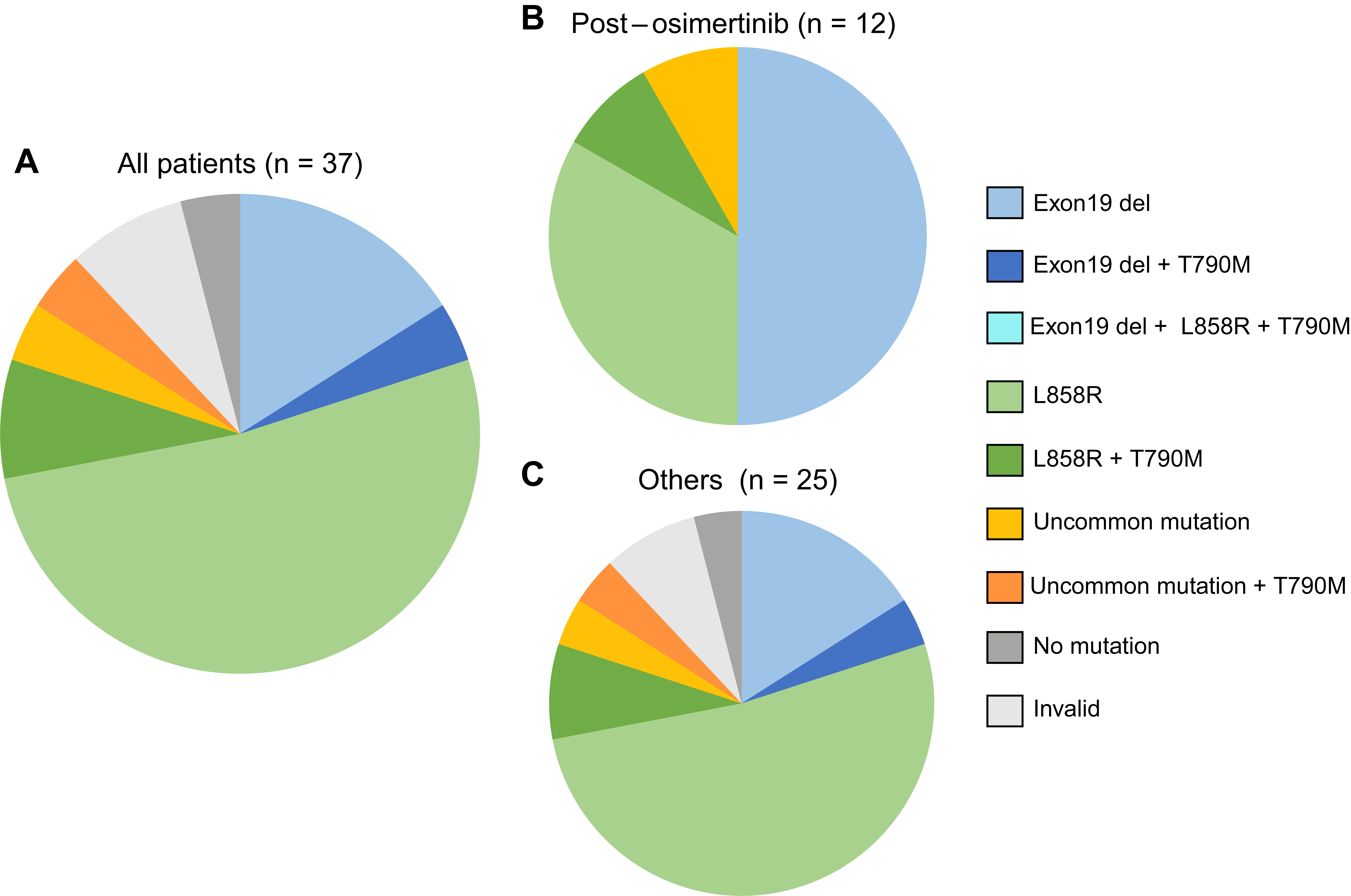


Supplemental Figure 2 Treatment Course and Seiral Rebiopsy results of Patient VGH034 who Harboring *EGFR* and *RET* Co-alteration After Osimeritnib Treatment Failure. CSF, cerebrospinal fluid; ddPCR, droplet digital polymerase chain reaction; NGS, next-generation sequencing; Bev, bevacizumab; ICI, immunocheckpoint inhibitors; CT, chemotheapy; Ram, ramucirumab.


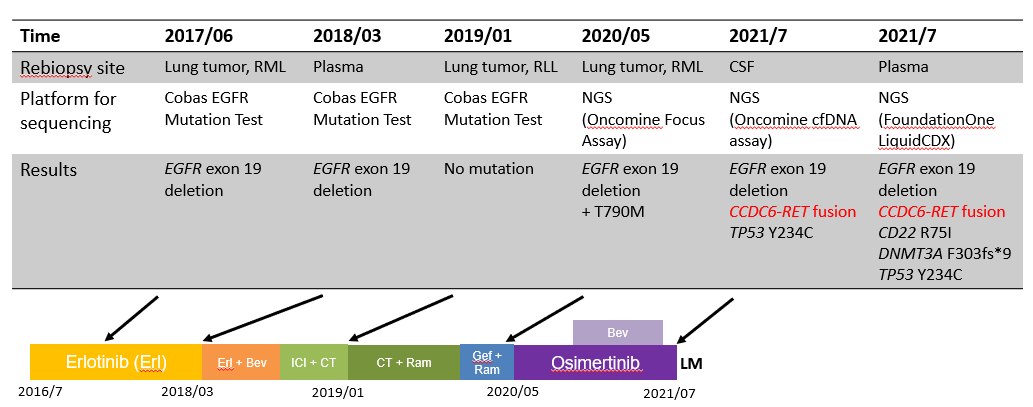

Supplement: Supplementary file 1 — Data S1. Supporting Information. [file TCA-14-1251-s001.docx]
